# Hypo-high density lipoproteinemia is a predictor for recurrent stroke during the long-term follow-up after revascularization in adult moyamoya disease

**DOI:** 10.3389/fneur.2022.891622

**Published:** 2022-07-26

**Authors:** Xiaofan Yu, Peicong Ge, Yuanren Zhai, Rong Wang, Yan Zhang, Dong Zhang

**Affiliations:** ^1^Department of Neurosurgery, Beijing Tiantan Hospital, Capital Medical University, Beijing, China; ^2^China National Clinical Research Center for Neurological Diseases, Beijing, China; ^3^Stroke Center, Beijing Institute for Brain Disorders, Beijing, China

**Keywords:** moyamoya disease, high density lipoprotein, stroke recurrence, hypo-high density lipoproteinemia, follow-up

## Abstract

**Objective:**

Previous studies have reported that hypo-high-density lipoproteinemia (HHDL) was an independent risk factor for the cerebrovascular event. However, the risk of HHDL for stroke recurrence in moyamoya disease (MMD) during long-term follow-up after revascularization remains poorly understood. We aim to investigate the association between HHDL and stroke recurrence in adult patients with MMD.

**Methods:**

A total of 138 adult patients with MMD were prospectively recruited from 1 July to 31 December 2019. After excluding 15 patients who did not meet the inclusion criteria, all the 123 patients were enrolled. Participants were grouped according to the stroke recurrence and HHDL presentation, respectively. Clinical data and laboratory examinations were compared by the statistical analysis. The Kaplan–Meier survival analysis was conducted to compare the stroke-free survival rates between participants with HHDL and those without. Univariate and multivariate logistic regression analyses were performed to identify independent factors of the neurological status. Univariate and multivariate Cox regression analyses were conducted to identify the predictors for the recurrent stroke.

**Results:**

Participants with recurrent stroke group showed a lower level of high-density lipoprotein (HDL) (*p* = 0.030). More participants in the recurrent stroke group had HHDL (*p* = 0.045). What is more, there was statistical significance in the Kaplan–Meier curve of stroke incidence between the normal HDL group and the HHDL group (log-rank test, *p* = 0.034). Univariate logistic analysis results showed that HHDL (OR 0.916, 95% CI 0.237–3.543; *p* = 0.899) and HDL (OR 0.729, 95% CI 0.094–5.648; *p* = 0.763) were not predictive factors for the neurological status. In the multivariate Cox regression analysis, diabetes (HR 4.195, 95% CI 1.041–16.899; *p* = 0.044), HDL (HR 0.061, 95% CI 0.006–0.626; *p* = 0.019), and HHDL (HR 3.341, 95% CI 1.110–10.051; *p* = 0.032) were independent risk factors for the recurrent stroke.

**Conclusions:**

Hypo-high-density lipoproteinemia might be a predictor or the potential therapeutic target for recurrent stroke during the long-term follow-up after revascularization in adult patients with MMD.

## Introduction

Moyamoya disease (MMD) is an unusual type of cerebrovascular disorder, associated with progressive stenosis of the intracranial internal carotid arteries and the aberrant vascular network in brain (1, 2). Also, MMD is the most common cause of stroke in children in China, Japan, and South Korea (3, 4).

Though stroke recurrence during long-term follow-up after revascularization is a rare situation, which may cause severe neurological dysfunction. It is necessary to investigate the relationship between stroke recurrence and MMD. Many studies have explored the risk factors for the postoperative stroke of MMD at the early phase (5–7). Nevertheless, there are few studies focusing on the risk factors of the stroke recurrence after surgical therapy during the long-term follow-up. One of our previous studies with 3–6 months of follow-up showed that hypertension and Suzuki stage were risk factors for the recurrent stroke in MMD (8). In addition, another retrospective study indicated that diabetes and posterior cerebral artery stenosis were predictors for stroke recurrence in patients with MMD aged 18–45 years (9). However, there is no consensus on the cause of stroke recurrence during the long-term follow-up in patients with MMD.

High-density lipoprotein (HDL), a common laboratory indicator, is composed of the lipids and proteins and the regulatory factors they carry. HDL exerts its anti-atherosclerotic effect by reversing cholesterol transport (10, 11). Previous studies have reported that hypo-high density lipoproteinemia (HHDL) was an independent risk factor for the cardiovascular disorders or cerebrovascular accidents (12, 13). A prospective cohort study among 267,500 participants indicated that the risks of ischemic and hemorrhagic stroke were higher when HDL was lower than 50 mg/dl during the long-term follow-up (14). Another prospective study based on the community cohorts also suggested that HHDL was associated with the risk of ischemic stroke over the follow-up of several years (15). In addition, a recent prospective cohort study found that HHDL was related to higher risks of total, ischemic, and hemorrhagic stroke during a median follow-up of 10 years (16). Nonetheless, the risk of HHDL for stroke recurrence in MMD during the long-term follow-up after revascularization remains elusive. In this research, we aimed to explore the association between HHDL and stroke recurrence by gathering the clinical and follow-up data of adult patients with MMD.

## Methods

### Study population

During 1 July to 31 December 2019, 138 adult participants with MMD were enrolled prospectively. Pediatric patients were not included in this study. In this study, we excluded 9 and 3 participants who did not accept surgical therapy or died due to comorbidities unrelated to MMD progression, respectively. Furthermore, three participants were lost to follow-up. Ultimately, 123 patients with MMD were enrolled in this research. Digital subtraction angiography (DSA) was used to diagnose MMD, according to the Japanese guidelines published in 2012 (17). The detailed study design and procedures have been described previously (18). The processes of this study were conducted on the basis of the guidelines of the Declaration of Helsinki. Moreover, this research was approved by the Ethics Committees of Beijing Tiantan Hospital, Capital Medical University. All the participants received and signed the informed consents.

### Data collection

The following clinical data were included: demographic information (age and sex), comorbidities, personal history (smoking and drinking), clinical characteristics (primary symptoms, heart rate, and blood pressure), laboratory examinations, and manifestations of the neuroimaging. Comorbidities were summarized into hypertension, diabetes mellitus, and thyroid disease. Suzuki stage and posterior cerebral artery involvement on the operative side was measured blindly through DSA by two neurosurgeons. Any divergence on the radiological manifestations was re-assessed by a third neurosurgeon. We evaluated the preoperative cerebral hemodynamic status of the participants by CT perfusion. Our previous study divided the hemodynamic status in the pre-stroke period into the following four stages (19): Stage I, time to peak (TTP) was delayed, mean transit time (MTT), regional cerebral blood flow (rCBF), and regional cerebral blood volume (rCBV) were normal; Stage II, TTP, and MTT were delayed, rCBF was normal, and rCBV was normal or slightly increased; Stage III, TTP, and MTT were delayed, cCBF was decreased, and rCBV was normal or slightly decreased; Stage IV, TTP, and MTT were delayed, rCBF and rCBV were decreased. The laboratory examinations concerning peripheral blood samples included glucose, creatinine (Cr), uric acid (UA), albumin (ALB), triglyceride (TG), total cholesterol (TC), HDL, low-density lipoprotein (LDL), apolipoprotein A (ApoA), apolipoprotein B (ApoB), and homocysteine (Hcy). Peripheral blood samples were gathered at 8:00 a.m. when patients with MMD had fasted for over 12 h. Serum Hcy levels above 15 μmol/L were defined as hyperhomocysteinemia (HHcy) (20). Serum HDL levels below 0.9 mmol/L were described as HHDL (21).

### Treatment and clinical outcomes

As described previously, three types of surgical treatment were conducted by neurosurgeons, namely, direct bypass, indirect bypass, and combined bypass. Direct bypass and combined bypass were performed for the most participants in our institution. When the superficial temporal artery or middle cerebral artery were too fragile to conduct artery anastomosis, we performed the indirect surgical bypass (7). As for the indication of revascularization, the clinical manifestation of patients should be considered first. The symptomatic hemisphere was given priority for the revascularization surgery. CT perfusion was taken into account for the patients who had no obvious symptom. The hemisphere with lower perfusion was treated first (22). The long-term outcome was determined through clinic visits and telephone interviews 24–35 months after discharge. Follow-up accidents concluded transient ischemia attack (TIA), ischemic stroke, hemorrhagic stroke, and loss of life. Recurrent stroke was regarded as a newly neurological deficiency that was persistent over 24 h, which was related to a new infarct or hemorrhage by using MRI or CT. The site and characteristics of the ischemic lesion were measured using the Oxfordshire Community Stroke Project (OCSP) system (23). OCSP subtypes included total anterior circulation infarcts (TACIs), partial anterior circulation infarcts (PACIs), posterior circulation infarcts (POCIs), and lacunar infarcts (LACIs). TACI refers to large anterior circulation infarcts with both the cortical and subcortical involvement, PACI refers to more restricted and predominantly cortical infarcts, POCI refers to infarcts associated with vertebrobasilar arterial territory, and LACI refers to infarcts confirmed to the territory of deep perforating arteries (24). The modified Rankin Scale (mRS) was to assess the neurological status at follow-up, with scores of 0–2 indicating a favorable neurological status and 3–6 representing an unfavorable neurological status. Assessment of the mRS score and stroke-free rate was performed by the two neurosurgical residents.

### Statistical analysis

All the statistical analysis was executed by using the IBM SPSS Statistics (version 22.0; IBM Corp.). The counts (percentages) were evaluated by the Pearson Chi-square test or the Fisher exact test. The continuous variables were assessed by *t*-test and the Mann–Whitney U test. The Kaplan–Meier (KM) survival analysis was conducted to compare the stroke-free survival rates between participants with HHDL or diabetes and those without. The identification of independent factors of the neurological status were evaluated by univariate and multivariate logistic regression analyses. Univariate and multivariate Cox regression analyses were performed to identify the predictors of the recurrent stroke. Statistical significance was regarded as *p* < 0.05 in a two-tailed test. Variables with *p* < 0.05 in univariate analysis were included in the multivariate analysis. Multivariate Cox regression analyses were adjusted for age, gender, glucose, Suzuki stage, primary symptom, and LDL.

## Results

### Baseline characteristics in participants with and without recurrent stroke

In total, 123 eligible patients with MMD, aged 19–61 years, were enrolled in the present study (39.02% males). The subjects were aged 39.11 ± 9.69 years (mean ± SD). The clinical and laboratory characteristics of the participants with and without stroke recurrence are shown in Table 1. Concerning the comorbidities, the incidence of diabetes was remarkably higher in the recurrent stroke group than in the non-recurrent stroke group (*p* = 0.022). No statistical differences in the primary symptom or personal history were presented between the two groups. Heart rate, blood pressure, BMI, surgical option, and the Suzuki stage also showed similarities between the recurrent group and the non-recurrent stroke group. Regarding laboratory examination, participants with the recurrent stroke group showed a lower level of HDL (*p* = 0.030). Moreover, more participants in the recurrent stroke group had HHDL (*p* = 0.045).

**Table 1:** Baseline characteristics of the participants with and without recurrent stroke.

**Characteristics**	**Overall (*****n*** = **123)**	**Recurrent stroke**		***P-*** **value**
		**Absent (*****n*** = **103)**	**Present (*****n*** = **20)**	
Age, y, mean (SD)	39.11 ± 9.69	38.41 ± 9.94	42.75 ± 7.52	0.067
Men (%)	48 (39.02)	40 (38.83)	8 (40.00)	0.922
Primary symptom (%)				0.145
Infarction	88 (71.54)	71 (68.93)	17 (85.00)	
Non-infarction	35 (28.46)	32 (31.07)	3 (15.00)	
Admission mRS (%)				0.488
0–2	115 (93.50)	97 (94.17)	18 (90.00)	
3–6	8 (6.50)	6 (5.83)	2 (10.00)	
Comorbidities (%)				
Hypertension	39 (31.71)	31 (30.10)	8 (40.00)	0.384
Diabetes	13 (10.57)	8 (7.77)	5 (25.00)	**0.022**
Thyroid disease	2 (1.63)	2 (1.94)	0 (0)	0.530
Personal history (%)				
Smoking	18 (14.63)	15 (14.56)	3 (15.00)	0.960
Drinking	15 (12.20)	14 (13.59)	1 (5.00)	0.283
Clinical feature, mean (SD)				
Heart rate, bpm	78.86 ± 8.48	78.41 ± 8.44	81.20 ± 8.52	0.179
SBP, mmHg	127.93 ± 14.48	128.05 ± 14.92	127.30 ± 12.27	0.833
DBP, mmHg	83.51 ± 9.14	83.20 ± 9.19	85.10 ± 8.96	0.398
BMI, kg/m^2^	25.12 ± 3.52	25.23 ± 3.60	24.60 ± 3.10	0.466
Laboratory results, median (IQR)				
WBC count, 10^9^/L	5.78 (5.06–6.93)	5.72 (5.06–6.96)	6.21 (4.75–6.79)	1.000
Lymphocyte count, 10^9^/L	1.97 (1.57–2.27)	1.95 (1.57–2.28)	1.99 (1.53–2.22)	0.926
PLT, 10^9^/L	240.00 (214.00–277.00)	238.00 (212.00–277.00)	248.00 (216.25–275.75)	0.805
Glucose, mmol/L	4.48 (4.18–4.98)	4.48 (4.18–4.98)	4.51 (4.19–4.95)	0.850
Creatinine, μmol/L	52.60 (45.40–64.90)	52.90 (45.40–64.90)	52.50 (44.10–65.53)	0.624
Uric acid, μmol/L	307.40 (243.20–376.80)	305.80 (243.20–376.80)	315.35 (240.50–393.03)	0.813
Albumin, g/L	42.40 (40.90–44.40)	42.40 (41.20–44.40)	41.70 (39.18–44.35)	0.222
Triglyceride, mmol/L	1.25 (0.92–1.84)	1.24 (0.92–1.80)	1.39 (1.01–1.95)	0.447
Total cholesterol, mmol/L	4.08 (3.52–4.65)	4.12 (3.58–4.60)	3.87 (3.27–4.93)	0.832
HDL, mmol/L	1.11 (0.91–1.30)	1.12 (0.96–1.33)	1.00 (0.78–1.22)	**0.030**
HHDL (%)	28 (22.76)	20 (19.42)	8 (40.00)	**0.045**
LDL, mmol/L	2.54 (2.05–3.05)	2.54 (2.07–3.04)	2.34 (1.93–3.52)	0.872
ApoA, g/L	1.25 (1.11–1.42)	1.25 (1.15–1.44)	1.27 (0.97–1.36)	0.208
ApoB, g/L	0.84 (0.73–0.98)	0.84 (0.74–0.96)	0.86 (0.70–1.08)	0.583
Homocysteine, μmol/L	12.49 (9.42–16.30)	12.49 (9.42–16.20)	12.45 (8.98–16.84)	0.834
HHcy (%)	35 (28.46)	29 (28.16)	5 (25.00)	0.773
Surgical option (%)				0.895
Indirect bypass	57 (46.34)	48 (46.60)	9 (45.00)	
Non-indirect bypass	66 (53.66)	55 (53.40)	11 (55.00)	
Suzuki stage (%)				0.260
I	4 (3.25)	4 (3.88)	0 (0)	
II	31 (25.20)	24 (23.30)	7 (35.00)	
III	53 (43.09)	46 (44.66)	7 (35.00)	
IV	24 (19.51)	22 (21.36)	2 (10.00)	
V	6 (4.88)	4 (3.88)	2 (10.00)	
VI	5 (4.07)	3 (2.91)	2 (10.00)	
The stage of pre-stroke period				0.847
Stage I	2 (1.63)	2 (1.94)	0 (0)	
Stage II	16 (13.01)	13 (12.62)	3 (15.00)	
Stage III	80 (65.04)	68 (66.02)	12 (60.00)	
Stage IV	25 (20.33)	20 (19.42)	5 (25.00)	
Posterior circulation involvement (%)	18 (14.63)	16 (15.53)	2 (10.00)	0.522
Discharge mRS (%)				0.738
0–2	113 (91.87)	95 (92.23)	18 (90.00)	
3–6	10 (8.13)	8 (7.77)	2 (10.00)	

SBP, systolic blood pressure; DBP, diastolic blood pressure; BMI, body mass index; HHDL, hypo-high density lipoproteinemia; HHcy, hyperhomocysteinemia; SD, standard deviation; IQR, interquartile range; mRS, modified Rankin scale.

Suzuki staging and posterior circulation involvement were defined on the operative side. The bold values mean P-value < 0.05.

### Clinical characteristics in the participants with and without HHDL

The clinical features of participants with and without HHDL are presented in Table 2. HHDL was observed in 28 participants (19 men and 9 women). The mean age of these participants was 42.18 ± 9.53 years. Compared with the normal HDL group, participants with HHDL were more likely to be men (*p* = 0.000). In the terms of primary symptom, 20 (71.43 %) participants were infarction and 8 (28.57 %) participants were non-infarction in HHDL group. As for comorbidities and personal history, participants with HHDL had higher incidences of diabetes (*p* = 0.000), smoking (*p* = 0.003), and drinking (*p* = 0.003) compared with the normal HDL group. Comparing to the normal HDL group, participants with HHDL showed a higher level of BMI (*p* = 0.050). In the terms of laboratory examinations, the incidences of UA (*p* = 0.013) and TG (*p* = 0.000) were significantly higher in participants with HHDL than those with normal HDL. Contrastingly, the incidences of TC (*p* = 0.002), LDL (*p* = 0.026), and ApoA (*p* = 0.000) were remarkably higher in the normal HDL group than in the HHDL group. Suzuki stage between the two groups also exhibited differences (*p* = 0.018).

**Table 2:** Clinical characteristics of the participants with and without HHDL.

**Characteristics**	**HHDL**		***P-*** **value**
	**Absent (*****n*** = **95)**	**Present (*****n*** = **28)**	
Age, y, mean(SD)	38.21 ± 9.61	42.18 ± 9.53	0.057
Men (%)	29 (30.53)	19 (67.86)	**0.000**
Primary symptom (%)			0.612
Infarction	63 (66.32)	20 (71.43)	
Non-infarction	32 (33.68)	8 (28.57)	
Admission mRS (%)			0.304
0–2	90 (94.74)	25 (89.29)	
3–6	5 (5.26)	3 (10.71)	
Comorbidities (%)			
Hypertension	30 (31.58)	9 (32.14)	0.955
Diabetes	5 (5.26)	8 (28.57)	**0.000**
Thyroid disease	2 (2.11)	0 (0)	0.439
Personal history (%)			
Smoking	9 (9.47)	9 (32.14)	**0.003**
Drinking	7 (7.37)	8 (28.57)	**0.003**
Clinical feature, mean (SD)			
Heart rate, bpm	79.48 ± 8.69	76.75 ± 7.49	0.134
SBP, mmHg	127.73 ± 15.21	128.61 ± 11.88	0.779
DBP, mmHg	83.66 ± 9.39	83.00 ± 8.39	0.737
BMI, kg/m^2^	24.79 ± 3.55	26.26 ± 3.22	0.050
Laboratory results, median (IQR)			
WBC count, 10^9^/L	5.76 (5.00–6.72)	6.04 (5.44–7.58)	0.175
Lymphocyte count, 10^9^/L	1.97 (1.57–2.27)	1.93 (1.56–2.45)	0.995
PLT, 10^9^/L	245.00 (214.00–277.00)	228.50 (206.00–271.50)	0.327
Glucose, mmol/L	4.43 (4.16–4.93)	4.72 (4.26–6.30)	0.061
Creatinine, μmol/L	51.60 (45.20–62.60)	58.05 (49.20–67.05)	0.183
Uric acid, μmol/L	302.60 (240.40–349.20)	371.20 (276.55–437.93)	**0.013**
Albumin, g/L	42.40 (41.20–44.50)	42.30 (39.33–43.83)	0.214
Triglyceride, mmol/L	1.14 (0.86–1.62)	1.91 (1.24–2.86)	**0.000**
Total cholesterol, mmol/L	4.21 (3.68–4.70)	3.57 (3.10–4.22)	**0.002**
LDL, mmol/L	2.63 (2.09–3.11)	2.20 (1.86–2.70)	**0.026**
ApoA, g/L	1.32 (1.21–1.47)	1.00 (0.93–1.10)	**0.000**
ApoB, g/L	0.84 (0.73–0.97)	0.86 (0.71–0.99)	0.937
Homocysteine, μmol/L	12.10 (9.25–16.20)	12.89 (9.78–17.28)	0.617
HHcy (%)	25 (26.32)	10 (35.71)	0.333
Surgical option (%)			0.992
Indirect bypass	44 (46.32)	13 (46.43)	
Non-indirect bypass	51 (53.68)	15 (53.57)	
Suzuki stage (%)			**0.018**
I	2 (2.11)	2 (7.14)	
II	20 (21.05)	11 (39.29)	
III	44 (46.32)	9 (32.14)	
IV	23 (24.21)	1 (3.57)	
V	3 (3.16)	3 (10.71)	
VI	3 (3.16)	2 (7.14)	
The stage of pre-stroke period			0.576
Stage I	2 (2.11)	0 (0)	
Stage II	14 (14.74)	2 (7.14)	
Stage III	61 (64.21)	19 (67.86)	
Stage IV	18 (18.95)	7 (25.00)	
Posterior circulation involvement (%)	14 (14.74)	4 (14.29)	0.953
Discharge mRS (%)			0.175
0–2	89 (93.68)	24 (85.71)	
3–6	6 (6.32)	4 (14.29)	

SBP, systolic blood pressure; DBP, diastolic blood pressure; BMI, body mass index; HHDL, hypo-high density lipoproteinemia; HHcy, hyperhomocysteinemia; SD, standard deviation; IQR, interquartile range; mRS, modified Rankin scale.

Suzuki staging and posterior circulation involvement were defined on the operative side. The bold values mean P-value < 0.05.

### Long-term clinical outcomes of participants with and without HHDL

**Figure 1:**
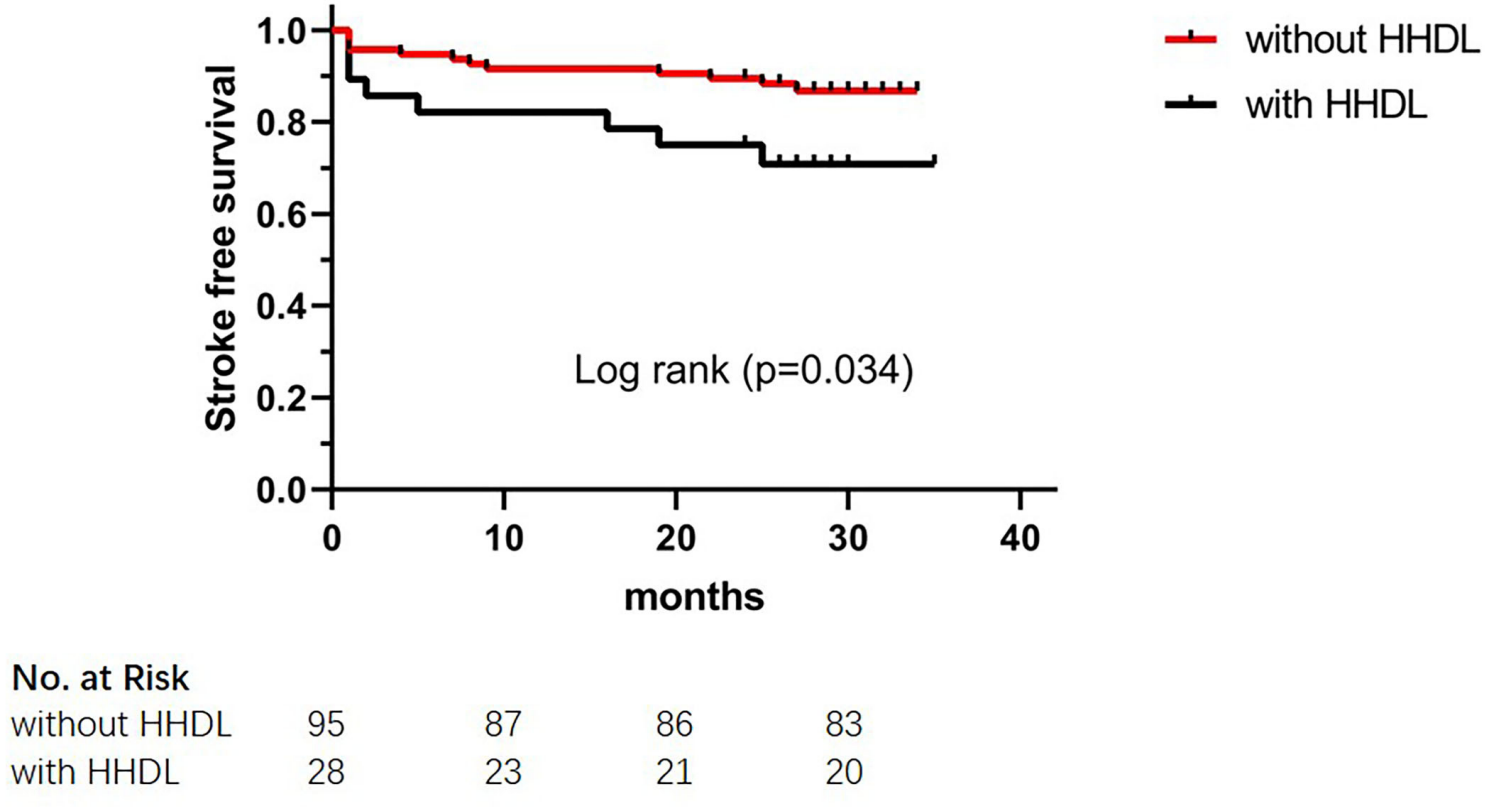
The Kaplan–Meier cumulative hazard curve for stroke recurrence comparing participants with HHDL and participants without HHDL.

**Figure 2:**
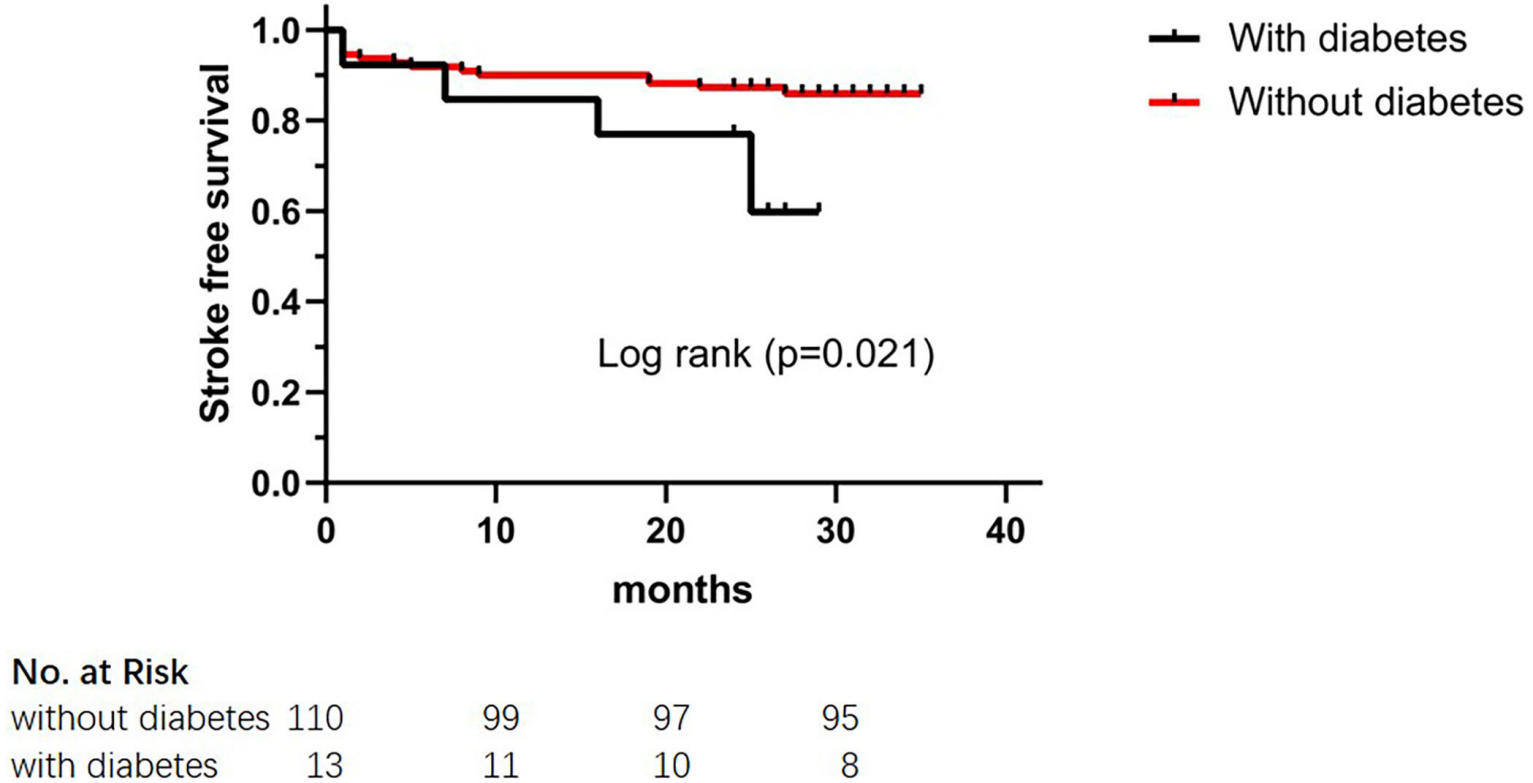
The Kaplan–Meier cumulative hazard curve for stroke recurrence comparing participants with diabetes and participants without diabetes.

The results of the long-term clinical outcomes analysis according to HHDL are shown in Table 3. After discharge, follow-up events during 28 months occurred more frequently in the HHDL group than in the normal HDL group (*p* = 0.025). However, no significant difference was displayed in the ischemic stroke, hemorrhagic stroke, and TIA (*p* > 0.05 for all). Functional outcomes (mRS score) did not show statistical differences between the two groups. What's more, there was statistical significance in the KM curve of stroke incidence between the normal HDL group and the HHDL group (log-rank test, *p* = 0.034) (Figure 1). As illustrated in Figure 2, the KM survival curve showed significant difference in the stroke incidence between the participants with diabetes and those without.

**Table 3:** Long-term outcomes of the participants with and without HHDL.

**Characteristics**	**Overall (*****n*** = **123)**	**HHDL**		***P-*** **value**
		**Absent (*****n*** = **95)**	**Present (*****n*** = **28)**	
Follow-up, median (IQR), m	28 (26–29)	28 (26–29)	27.5 (26–29)	0.648
Follow-up events	58 (47.15)	32 (33.68)	16 (57.14)	**0.025**
Ischemic stroke	17 (13.82)	10 (10.53)	7 (25.00)	0.051
Hemorrhagic stroke	3 (2.44)	2 (2.11)	1 (3.57)	0.659
TIA	28 (22.76)	20 (21.05)	8 (28.57)	0.404
Neurological status (mRS score)				0.899
mRS score 0–2	109 (88.62)	84 (88.42)	25 (89.29)	
mRS score 3–5	14 (11.38)	11 (11.58)	3 (10.71)	
Death^*^	3 (2.44)			

HHDL, hypo-high density lipoproteinemia; mRS, modified Rankin scale; IQR, interquartile range.

* Three patients died due to comorbidities unrelated to moyamoya disease progression. The bold values mean P-value < 0.05.

### Predictive factors of the neurological status

Predictive factors of the neurological status were also evaluated in this study. As demonstrated in Table 4, univariate logistic regression indicated that diabetes (OR 4.444, 95% CI 1.158–17.063; *p* = 0.030) and Suzuki stage (OR 1.853, 95% CI 1.134–3.028; *p* = 0.014) were related to the neurological status. It also could be found from the univariate logistic regression that HHDL (OR 0.916, 95% CI 0.237–3.543; *p* = 0.899) and HDL (OR 0.729, 95% CI 0.094–5.648; *p* = 0.763) were not related to the neurological status. Multivariate logistic regression analysis showed that the Suzuki stage (OR 1.779, 95% CI 1.085–2.917; *p* = 0.022) was significantly related to the neurological status. Diabetes (OR 4.036, 95% CI 0.974–16.732; *p* = 0.054) was not a predictive factor of the neurological status in the multivariate regression analysis.

**Table 4:** Univariate and multivariate logistic analysis for neurological status.

**Variables**	**Univariate analysis**			**Multivariate analysis**		
	**OR**	**95% CI**	***P*** **-value**	**OR**	**95% CI**	***P*** **-value**
Age	1.034	0.975–1.096	0.262			
Men	1.173	0.368–3.736	0.788			
Primary symptom						
Infarction	Ref	Ref	Ref			
Non-infarction	0.994	0.290–3.406	0.992			
Admission mRS	2.861	0.518–15.793	0.228			
Comorbidities						
Hypertension	0.553	0.145–2.107	0.385			
Diabetes	4.444	1.158–17.063	**0.030**	4.036	0.974–16.732	0.054
Personal history						
Smoking	1.709	0.427–6.848	0.449			
Clinical feature						
Heart rate, bpm	1.003	0.940–1.071	0.921			
SBP	1.006	0.969–1.045	0.738			
DBP	1.039	0.976–1.107	0.230			
BMI	0.981	0.836–1.151	0.817			
Laboratory examination						
WBC count	0.887	0.608–1.292	0.531			
Lymphocyte count	0.673	0.239–1.897	0.454			
PLT	0.991	0.979–1.004	0.161			
Glucose	1.158	0.858–1.562	0.338			
Creatinine	0.970	0.926–1.017	0.214			
Uric acid	0.996	0.990–1.002	0.164			
Albumin	0.897	0.746–1.078	0.246			
Triglyceride	0.766	0.360–1.628	0.488			
Total cholesterol	0.825	0.408–1.671	0.594			
HDL	0.729	0.094–5.648	0.763			
HHDL	0.916	0.237–3.543	0.899			
LDL	0.842	0.391–1.812	0.659			
ApoA	0.581	0.054–6.246	0.654			
ApoB	0.438	0.019–10.143	0.606			
Homocysteine	0.995	0.947–1.045	0.839			
HHcy	1.463	0.453–4.719	0.524			
Surgical option						
Indirect bypass	Ref	Ref	Ref			
Non-indirect bypass	1.172	0.381–3.605	0.781			
Suzuki stage	1.853	1.134–3.028	**0.014**	1.779	1.085–2.917	**0.022**
The stage of pre-stroke period	0.468	0.179–1.228	0.123			
Posterior circulation involvement	2.714	0.749–9.842	0.129			
Discharge mRS	3.974	0.897–17.610	0.069			

SBP, systolic blood pressure; DBP, diastolic blood pressure; BMI, body mass index; HHDL, hypo-high density lipoproteinemia; HHcy, hyperhomocysteinemia; mRS, modified Rankin scale. The bold values mean P-value < 0.05.

### Predictive factors of the recurrent stroke

As shown in Table 5, we identified diabetes (HR 3.078, 95% CI 1.116–8.492; *p* = 0.030), HDL (HR 0.121, 95% CI 0.018–0.815; *p* = 0.030), HHDL (HR 2.526, 95% CI 1.031–6.187; *p* = 0.043) were predictive for the recurrent stroke in the univariate Cox regression analysis. In the multivariate analysis, diabetes (HR 4.195, 95% CI 1.041–16.899; *p* = 0.044), HDL (HR 0.061, 95% CI 0.006–0.626; *p* = 0.019), HHDL (HR 3.341, 95% CI 1.110–10.051; *p* = 0.032) were independent risk factors for the recurrent stroke. Multivariate analyses were adjusted for age, gender, glucose, Suzuki stage, primary symptom, and LDL.

**Table 5:** Univariate and multivariate Cox regression analysis for recurrent stroke.

**Variables**	**Univariate analysis**			**Multivariate analysis** ^*^		
	**HR**	**95% CI**	***P*** **-value**	**HR**	**95% CI**	***P*** **-value**
Age	1.043	0.997–1.091	0.066			
Men	0.962	0.393–2.353	0.932			
Primary symptom						
Infarction	Ref	Ref	Ref			
Non-infarction	0.662	0.240–1.822	0.424			
Admission mRS	0.569	0.132–2.455	0.450			
Comorbidities						
Hypertension	1.473	0.602–3.605	0.396			
Diabetes	3.078	1.116–8.492	**0.030**	4.195	1.041–16.899	**0.044**
Personal history						
Smoking	1.000	0.293–3.411	0.999			
Clinical feature						
Heart rate, bpm	1.031	0.985–1.079	0.191			
SBP	0.996	0.967–1.027	0.821			
DBP	1.022	0.973–1.073	0.390			
BMI	0.952	0.839–1.080	0.444			
Laboratory examination						
WBC count	0.902	0.671–1.212	0.493			
Lymphocyte count	0.975	0.449–2.121	0.950			
PLT	1.001	0.993–1.010	0.800			
Glucose	1.069	0.833–1.373	0.600			
Creatinine	0.990	0.957–1.025	0.580			
Uric acid	1.001	0.997–1.006	0.544			
Albumin	0.862	0.739–1.006	0.060			
Triglyceride	1.371	0.931–2.018	0.110			
Total cholesterol	0.975	0.563–1.691	0.929			
HDL	0.121	0.018–0.815	**0.030**	0.061	0.006–0.626	**0.019**
HHDL	2.526	1.031–6.187	**0.043**	3.341	1.110–10.051	**0.032**
LDL	1.100	0.615–1.967	0.747			
ApoA	0.203	0.027–1.522	0.121			
ApoB	2.582	0.250–26.653	0.426			
Homocysteine	0.990	0.947–1.035	0.653			
HHcy	1.086	0.417–2.825	0.866			
Surgical option						
Indirect bypass	Ref	Ref	Ref			
Non-indirect bypass	1.080	0.448–2.607	0.863			
Suzuki stage	1.131	0.754–1.695	0.552			
The stage of pre-stroke period	1.142	0.566–2.307	0.710			
Posterior circulation involvement	0.602	0.140–2.597	0.497			
Discharge mRS	0.758	0.176–3.270	0.710			

SBP, systolic blood pressure; DBP, diastolic blood pressure; BMI, body mass index; HHDL, hypo-high density lipoproteinemia; HHcy, hyperhomocysteinemia; mRS, modified Rankin scale.

Suzuki staging and posterior circulation involvement were defined on the operative side.

* Analyses were adjusted for age, gender, glucose, Suzuki stage, primary symptom, and LDL. The bold values mean P-value < 0.05.

### OCSP classification of the recurrent ischemic stroke

We utilized the OCSP classification to classify 17 participants who developed the recurrent ischemic stroke. As illustrated in Table 6, 6 cases in the HHDL group and 4 cases in the diabetes group were classified as LACI among the 17 participants with the recurrent ischemic stroke. Only one participant in each of the two groups was classified as PACI.

**Table 6:** OCSP classification of recurrent ischemic stroke.

**Group**	**TACI**	**PACI**	**POCI**	**LACI**
With HHDL	0	1	0	6
Without HHDL	1	0	1	8
With diabetes	0	1	0	4
Without diabetes	1	0	1	10

The bold values mean P-value < 0.05.

## Discussion

The purpose of this prospective study involving adult patients with MMD was to explore the relationship between HHDL and the stroke recurrence. This study showed that that HHDL was significantly related to an increased risk of the recurrent stroke. Contrastingly, HHDL was not a predictive factor of an unfavorable neurological status over a follow-up of 24–35 months. The results of our prospective study may reveal a potential predictor of stroke recurrence during the long-term follow-up after revascularization in patients with MMD.

Hypo-high-density lipoproteinemia is a risk factor for the cardiovascular and cerebrovascular events (12, 14). Increasing evidences have indicated that an association between HHDL and increased risk of stroke. Several studies have reported that HHDL might increase the risk of ischemic stroke during the long-term follow-up (14, 15). In addition, HHDL was found to be a risk factor for the hemorrhagic stroke over the follow-up of several years (14). In this study, we also found that HHDL was remarkably associated with a higher risk of recurrent stroke during the long-term follow-up in patients with MMD. Although how HHDL leads to the recurrent stroke of MMD is still unclear, some hypothesis may help us to understand this process. HDL, or “good” cholesterol, is a heterogeneous group of lipid-protein complexes. The major biological function of HDL is to transport cholesterol from the extrahepatic tissues to the liver, thus, exerting an anti-atherosclerotic effect (25). What is more, another crucial biological role of HDL is protection against inflammation (26). Inflammation plays a crucial role in the initiation and progress of atherosclerosis, which could cause stroke (27). Understandably, the anti-atherosclerotic effect of the HHDL group was weakened, leading to the recurrent stroke. Furthermore, several preclinical and clinical studies have demonstrated that HDL could promote angiogenesis (28–30). We speculated that participants in the normal HDL group had better postoperative collateral formation than participants with HHDL. Thus, according to this theory, the HHDL group was more likely to have recurrent stroke than the normal HDL group. Arregui et al. illustrated the relationship between HDL and ischemic stroke from a genetic perspective. Their finding suggested that both plasma levels of HDL and ischemic stroke were associated with rs2943634 (2q36.3) genetic polymorphism (31). Therefore, it is expected to further investigate the association between recurrent stroke and HDL in patients with MMD from the genetic perspective in the future. To sum up, HHDL might be a promising predictor of the recurrent stroke during the long-term follow-up after revascularization in patients with MMD. Physicians should pay appropriate attention to plasma levels of HDL during the long-term follow-up of patients with MMD.

Several previous studies have indicated an association between the diabetes and recurrent stroke (32–34). In addition, a recent retrospective study identified diabetes as the independent risk factor of stroke recurrence after revascularization in adult patients with MMD (9). The same conclusion was reached in this study. In addition, a large number of epidemiological evidences have suggested that lower HDL level was correlated with the higher risk of diabetes (35–37). We also noted a higher proportion of diabetes in the HHDL group in this study. The association between low HDL levels and diabetes mellitus is complicated and has not been elucidated fully. Thus, further studies are needed to confirm the relationship among HHDL, diabetes and recurrent stroke. Contrastingly, HHDL was not related to a higher risk of an unfavorable neurological status. So, HHDL cannot be used to evaluate the long-term functional outcomes of patients with MMD. Moreover, neither functional outcomes nor recurrent stroke was associated with the Suzuki stage in a retrospective study (9). Suzuki stage was a predictor for an unfavorable neurological status but was not associated with the recurrent stroke in this prospective study. In conclusion, Suzuki stage could predict the long-term functional outcomes to some extent, but further clinical evidence is needed to confirm this point. Furthermore, our data suggested that preoperative hemodynamic status was not associated with the long-term outcomes or stroke recurrence. The Berlin grading system, proposed by Czabaka et al., consists of DSA, MRI, and cerebrovascular reserve capacity (38). Some studies have shown that the Berlin grading system could stratify preoperative clinical severity (39, 40). In addition, perioperative complications and long-term functional outcomes were associated with the Berlin grading system (39, 40). However, acetazolamide challenge test was not performed on the enrolled patients in this study. We will confirm this hypothesis in the future studies. We utilized the OCSP classification to classify 17 participants who developed the recurrent ischemic stroke. The vast majority of participants in both the HHDL group and diabetes groups were classified as LACI. Therefore, we have reason to believe that recurrent ischemic stroke in this study was mainly related to moyamoya disease rather than atherosclerosis.

To sum, this study has illustrated the significant role of HHDL in stroke recurrence during the long-term follow-up after revascularization in adult patients with MMD. HHDL could be a potential therapeutic target, which indicated that recurrent stroke might be prevented by monitoring HHDL. Furthermore, diabetes could also be regarded as a risk factor for the recurrent stroke in adult patients with MMD.

## Limitations

This study had several limitations. First, the sample size of this prospective study was relatively limited; second, our clinical data from a single-center, the results need to be validated in the multiple centers; third, recurrent stroke during the long-term follow-up after revascularization is a rare situation. Our results may be biased to some extent due to the low incidence; fourth, the reproducibility of this study was poor; fifth, only the Chinese adult patients with MMD included, these findings could not be generalized to other ethnicities.

## Conclusions

In general, HHDL was significantly associated with the recurrent stroke during the long-term follow-up after revascularization in adult patients with MMD. Thus, HHDL maybe a predictor or target of drug intervention for the recurrent stroke during the long-term follow-up after revascularization. The monitoring of HDL levels and the mechanism of HHDL could be the direction of the future research.

## Data availability statement

The raw data supporting the conclusions of this article will be made available by the authors, without undue reservation.

## Ethics statement

The studies involving human participants were reviewed and approved by IRB of Beijing Tiantan Hospital. The patients/participants provided their written informed consent to participate in this study. Written informed consent was obtained from the individual(s) for the publication of any potentially identifiable images or data included in this article.

## Author contributions

Conceptualization and methodology: PG and DZ. Data curation and writing—original draft: XY. Visualization, investigation, software, and validation: YZ. Supervision: RW. All authors contributed to the article and approved the submitted version.

## Conflict of interest

The authors declare that the research was conducted in the absence of any commercial or financial relationships that could be construed as a potential conflict of interest.

## Publisher's Note

All claims expressed in this article are solely those of the authors and do not necessarily represent those of their affiliated organizations, or those of the publisher, the editors and the reviewers. Any product that may be evaluated in this article, or claim that may be made by its manufacturer, is not guaranteed or endorsed by the publisher.
